# Reply: Assessing health risks of complementary alternative medicines in cancer patients

**DOI:** 10.1038/sj.bjc.6602046

**Published:** 2004-07-13

**Authors:** U Werneke, J Earl, C Seydel, O Horn, P Crichton, D Fannon

**Affiliations:** 1 Department of Psychiatry, Homerton Hospital, East Wing, Homerton Row, London E9 6SR, UK

**Sir,**

Drug information services such as the Chinese Medicine Advisory Service at the Medical Toxicology Unit at Guy's and St Thomas' Hospital Trust make an important contribution to the safety management of patients taking complementary alternative medicines (CAMs). We are aware that these services exist and increased resort to the service they offer is needed. Our proposal is that doctors will need to devote time to discussing CAM use in outpatient clinics although the complexities of side effects and interactions may require clinics which are run jointly with a local medicines information and toxicology services (Werneke *et al*, 2004). Such work may not be feasible in routine outpatient clinics where patients may be seen only briefly. Joint clinics would not only address drug safety concerns but also the patients' motivation for opting for CAMs, for instance to gain a greater degree of control over their illness and its treatment, and thereby to regain control over their lives (Sparber *et al*, 2000).

We agree with Shia *et al* that herbal remedies may have beneficial synergistic effects with conventional therapies. However, patients may take CAMs for a variety of reasons including reduction of cytotoxicity and its associated side effects rather than increasing it. This is highlighted by an e-mail we received in response to out study from a naturopath who asserted:’ …you will find that more people die of the chemotherapy than the cancer itself. Therefore if the herbs are making the chemo less effective this could be a beneficial thing…’. Thus, there is a need to work out and implement an individualised treatment plan for each patient wishing to use CAMs, and this may be beyond the scope of a drug information service.

Shia *et al* suggest prospective studies to evaluate the interactions between conventional and complementary medicines. However, such studies may be difficult to conduct if there are reasons to suspect potentially serious interactions or a significant reduction in efficacy of the conventional treatment, which could lead to a reduction in survival time. It may not be ethically acceptable to opt for ‘watchful waiting’ in such cases, and one may wish to err on the side of caution. In view of this, we recommended stopping CAMs in some cases while monitoring for adverse effects in others. Prospective studies could be further complicated by the fact that many patients take combinations of remedies rather than one substance only, which may not always make it possible to attribute an adverse reaction unambiguously to one agent.

In consequence, we feel that the research on and management of CAMs should be integrated into the conventional cancer care to provide a truly holistic approach and to enable clinicians to adapt CAMs and conventional therapies to patients' individual requirements and to take account of changes in the clinical course whenever they occur.
